# Variance in translational fidelity of different bacterial species is affected by pseudouridines in the tRNA anticodon stem-loop

**DOI:** 10.1080/15476286.2022.2121447

**Published:** 2022-09-11

**Authors:** Karl Jürgenstein, Mari Tagel, Heili Ilves, Margus Leppik, Maia Kivisaar, Jaanus Remme

**Affiliations:** Institute of Molecular and Cell Biology, University of Tartu, Tartu, Estonia

**Keywords:** tRNA, pseudouridine, translation accuracy, frameshift error, renilla-firefly luciferase fusion

## Abstract

Delicate variances in the translational machinery affect how efficiently different organisms approach protein synthesis. Determining the scale of this effect, however, requires knowledge on the differences of mistranslation levels. Here, we used a dual-luciferase reporter assay cloned into a broad host range plasmid to reveal the translational fidelity profiles of *Pseudomonas putida, Pseudomonas aeruginosa* and *Escherichia coli*. We observed that these profiles are surprisingly different, whereas species more prone to translational frameshifting are not necessarily more prone to stop codon readthrough. As tRNA modifications are among the factors that have been implicated to affect translation accuracy, we also show that translational fidelity is context-specifically influenced by pseudouridines in the anticodon stem-loop of tRNA, but the effect is not uniform between species.

## Introduction

Translation is a well-conserved process responsible for accurately synthesizing proteins based on genetic information and necessary for all cellular processes. As demand for proteins in a cell is high, trade-off between speed and accuracy leads to some aberrations in nascent peptides, either because of errors during decoding or failure to maintain the correct reading frame. Missense error frequency during translation is usually lower than 1/1000 [1–3], whereas frameshift errors are by two orders of magnitude less frequent [4]. However, misincorporation of amino acids and programmed frameshift can occur in special cases at frequency as high as 10–20% [2,5]. Translation accuracy has been measured at quantitative level mostly in few model species, such as *E. coli* and *Saccharomyces cerevisae,* and it is evident that it varies significantly between different organisms [2]. Differences in translational fidelity can shape the proteome and contribute to the adaptation with stressful conditions, however, according to our knowledge, no data for systematic comparison of error frequency of different bacterial species is available.

tRNA is involved in several cellular processes in addition to its central role in translation of genetic message. During translation tRNA molecules transfer activated amino acids from aminoacyl-tRNA synthetases to the ribosome for the mRNA directed protein synthesis. Non-canonical roles of tRNAs beyond translation involve biosynthesis of amino acids, modification of peptides and lipids, nucleotide alarmone synthesis, regulation of gene expression, etc., and the list of tRNA mediated cellular functions is still growing [6]. tRNA is the most heavily modified RNA molecule in bacteria; about 10% of its nucleotides contain chemical modifications [7]. The modifications are often found at certain regions, e.g. anticodon stem and loop structure (ASL), and have been implicated to determine codon reading specificity and translation fidelity [8–11].

A large variety of modified nucleotides are found at position 34 (first position of anticodon) of elongator tRNAs. Nucleotide at the position 37, just 3´ of anticodon, contains complex modifications, which are involved in stabilizing codon – anticodon pairing and in improvement of reading frame maintenance [10,12]. Another set of modifications bracketing the anticodon loop are pseudouridines (positions 32, 38–40). These understudied Ψs are found in a subset of tRNA species and are introduced into tRNAs by pseudouridine synthases. Deletion of enzyme RluA gene leads to loss of isomerization of U746 of 23S rRNA and U32 in four *E. coli* tRNA species [13]. Uridine residues at tRNA positions 38–40 are not isomerized in about half of tRNA species in the absence of TruA [14,15]. Similar loss of uridine isomerization around tRNA ASL is observed in *P. putida* upon deletion of RluA and TruA genes [16]. Both TruA and RluA have been suggested to have a tRNA chaperone activity [17]. In spite of the possible double function both genes are nonessential in *E. coli* [18]. Loss of TruA function leads to inefficient reading of consecutive codons by its substrate tRNAs [19] and decreased mistranslation at His codons under histidine starvation [20]. Increased frameshifting for Ψ38-deficient tRNA^Leu^ has been observed [21]. These results indicate that TruA has a function in maintaining translational fidelity. Systematic analysis of TruA in respect of codon reading accuracy and reading frame maintenance is still missing.

Structural and comparative studies have revealed that the first and the last nucleobase of the anticodon loop (bases 32 and 38) tend to form a non-Watson-Crick pair with a single hydrogen bond [22]. This interaction can be facilitated by a pseudouridine, as it forms more stable base pairs and stacking interactions compared to the unmodified uridine [23–25]. Interaction between bases 32 and 38 is transiently lost during +1 frameshifting [26,27]. Thus, pseudouridine at position 38/39 (isomerized by TruA) or 32 (isomerized by RluA) could help to keep codon reading frame by local stabilization of anticodon loop structure. If this is true, deletion of TruA or RluA is expected to increase frameshifting frequency. We aimed to test this possibility by combining TruA or RluA deletion strains of three different bacterial species with a series of frameshift and stop codon reporters.

So far, *E. coli* has served as the primary model in investigating molecular mechanisms affecting translation accuracy in bacteria. The genus *Pseudomonas* represents one of the largest group of bacteria that are known for their versatility and adaptability in hostile and fluctuating habitats [28]. *Pseudomonas putida*, e.g., is found mostly in temperate soil and water habitats. The metabolic versatility and high tolerance to toxic and harsh conditions makes *P. putida* attractive for biotechnological applications such as biodegradation of environmental pollutants and synthesis of added-value chemicals [29–31]. *Pseudomonas aeruginosa*, on the other hand, although found widely in the environment, is mostly known as an opportunistic hospital-acquired human pathogen, responsible for both chronic and acute infections [32,33].

Recently we have demonstrated that the tRNA modification enzymes TruA and RluA in soil bacterium *P. putida* possess similar targets as have been shown in *E. coli* and the absence of said enzymes increases mutation frequency in *P. putida* via lack of tRNA pseudouridylation [16,34]. Loss of translation fidelity has been suggested to affect error frequency of DNA replication [35–37]. In this respect we wanted to analyse whether the deletion of pseudouridine synthases TruA and RluA influences the translation fidelity and frameshifting in particular. Comparative analysis of translation error frequency was performed in three bacterial species *Pseudomonas putida, Pseudomonas aeruginosa* and *Escherichia coli* using the same reporter constructs encoded by a shuttle vector.

## Results

### Translational accuracy within the same genetic context differs between Pseudomonas putida, Pseudomonas aeruginosa and Escherichia coli

**Figure 1.**
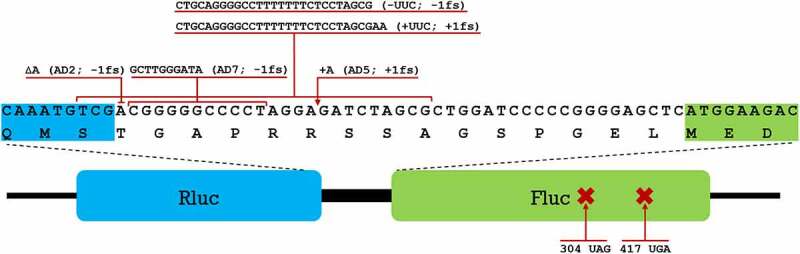
Different constructs of the dual-luciferase assay system used to measure the translational fidelity. Rluc (blue) and Fluc (green) form a fusion protein connected by a short linker region. Nucleotide and amino acid sequence of the linker region is shown with adjacent regions of Rluc and Fluc highlighted. Different insertions and deletions introduced into the system to measure frameshifting are shown with red lines above the sequence, premature stop codons with their positions are shown below.

To estimate error frequency of translation in three bacterial species, we exploited a dual-luciferase assay system. This assay is based on a fusion protein consisting of two luciferases (Rluc and Fluc). Test sequence containing slippery signal is inserted between the two cistrons. In this way Fluc is synthesized only after a frameshift event has occurred during mRNA translation. Thanks to the sensitivity and internal control of the system it is an efficient tool for analysis of translation accuracy at various sequence contexts. This reporter (Figure 1), with its different derivatives carrying mutations in the linker-region to detect translational frameshifting or premature stop codons in the *fluc* gene to detect stop codon readthrough [38], was inserted into a broad host range plasmid pSEVA/lacItac [16] to allow its simultaneous usage in three bacterial species: *P. putida, P. aeruginosa* and *E. coli*.

Using a set of plasmids encoding different derivatives of the Rluc-Fluc assay system, we measured the translational fidelity in seven different genetic contexts (Figure 1) to compare the basic translation fidelity profiles of the three bacterial species. In total there were 5 frameshifts, of which AD2 (−1 FS), AD5 (+1 FS) and AD7 (−1FS) have been described previously [38], whereas slippery site containing -UUC (−1FS) and +UUC (+1FS) were constructed in this work. Stop codon readthrough was observed using plasmids where *fluc* gene contained premature stop codons at positions 304 (304 UAG) and 417 (417 UGA) [38]. Detailed explanation with corresponding codons and tRNAs targeted by TruA and RluA within each of these contexts is available in supplementary materials (Tables S1-S8).

**Figure 2.**
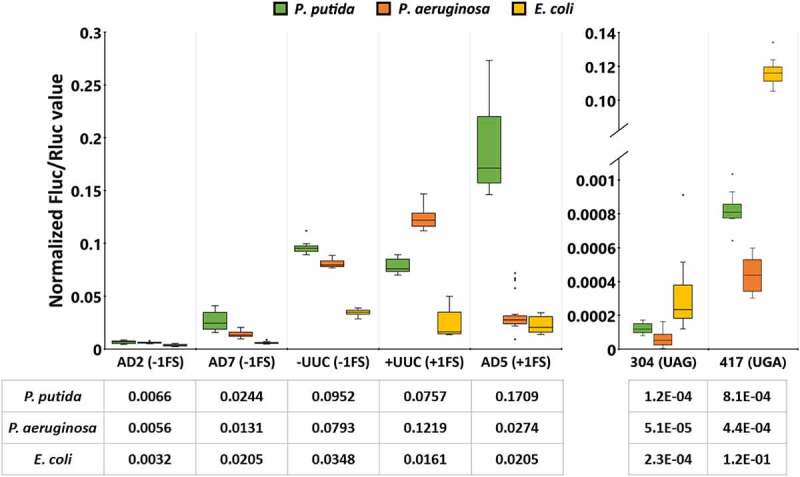
Translation errors among different genetic contexts (shown on the x axis) in wild-type strains of three bacterial species: Pseudomonas putida strain PaW85 (depicted in green), Pseudomonas aeruginosa strain PAO1-L (Orange) and Escherichia coli strain MG1655 (yellow). For the frameshift events both the name used to refer to specific context and the direction (either −1 or +1 frameshift) is shown; for the stop codon readthrough events the position of the codon within the assay system that has been mutated to produce a premature stop codon and the resulting stop codon are shown. In all cases n ≥ 11. Boxplots show the Fluc/Rluc values normalized against the Fluc/Rluc value of an unmutated (wild-type) test system in the corresponding species. Line in the box denotes the median value (also shown in table under the graph), the upper and lower borders of the box represent first and third quartile, the whiskers show the non-outlier range and circles are outliers.

Results of the dual-luciferase assays in wild-type bacterial species, as the basic translation fidelity profiles of the three bacterial species, are shown in Figure 2. Firstly, it is evident that error profiles of the three species are notably different. Translation error frequency in *P. putida* cells varies significantly, depending on the codon context and the type of error-prone sequence. Out of three −1 FS reporters UUC exhibited significantly higher frameshifting as compared to AD2 and AD7. +1 FS in the AD5 context was the most frequent of all frameshift events tested, occurring in over 15% of the cases when Rluc was translated, about two times or more frequently than other frameshift errors. −1 FS reporter AD2 has similar sequence as AD5 except an A is deleted while in AD5 an A is inserted (Figure 1). FS frequency was more than an order of magnitude higher in AD5 as compared to AD2. In contrast, at the slippery sequence UUC, −1 and +1 frameshift occurred with nearly equal frequency. UGA stop codon was suppressed more often than UAG, however, compared to translational frameshifting, stop codon readthrough levels were orders of magnitude lower, at 0.08% (UGA) and 0.01% (UAG) compared to 0.6% (AD2, −1FS) to 17% (AD5, +1FS).

In *P. aeruginosa*, translational frameshifting occurred with low frequency except at the slippery sequence (UUC) when +1 frameshifting took place at a higher frequency – around 12% of all translation events, compared to 8% in the case of −1 FS. Translational readthrough at stop codons was once again very rare, with readthrough event taking place at a frequency of 1/2500 (UGA) or 1/20,000 (UAG) (Figure 2).

In *E. coli*, translational frameshifting stayed below 5% in all reporters. UGA stop codon suppression level was around 12%, 500 times higher than UAG stop codon suppression (Figure 2). Compared to *P. putida* and *P. aeruginosa*, UGA suppression was 148- or 272-fold higher, respectively.

Although the translational machinery is well conserved in all prokaryotes, significant variation still exists between species regarding errors made during protein synthesis. *P. putida* appeared to be slightly more error-prone than *P. aeruginosa* and *E. coli*, at least in the investigated contexts, however no clear and universal patterns emerged. Both *Pseudomonas* species exhibited frequent frameshifting at UUC slippery sites in both directions. Interestingly, while *E. coli* tended to be more frameshift-resistant across all studied contexts, it exhibited higher stop codon readthrough rate on both UAG and especially on UGA stop codons, than in *P. putida* and *P. aeruginosa*.

### Effect of tRNA anticodon stem-loop pseudouridines on mistranslation

In order to detect whether the lack of pseudouridines in the ASL has an effect on translational fidelity, pseudouridine synthases-deficient ∆*truA* and ∆*rluA* strains of *P. putida, P. aeruginosa* and *E. coli* were analysed with the Rluc-Fluc assay system.

**Figure 3.**
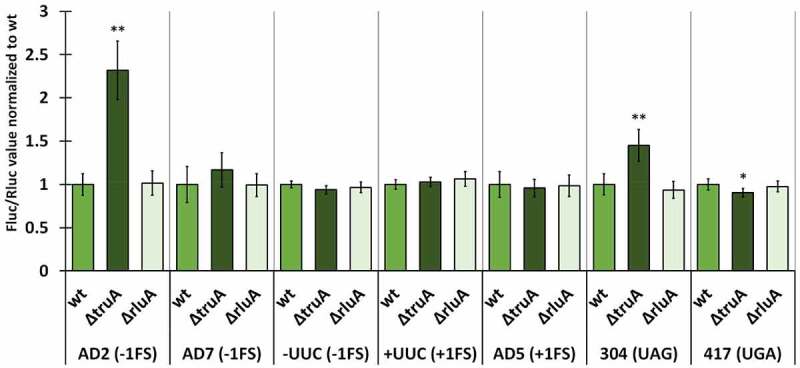
Effect of pseudouridines on translation error frequency using ΔtruA and ΔrluA strains relative to wild-type in P. putida PaW85. Deletion strains have been normalized against the wild-type strain within each genetic context. Error bars represent CI 95%, ‘*’ indicates p-value <0.05, ‘**’ indicates p-value <0.001 compared to the respective wild-type, n ≥ 11.

We normalized the Fluc/Rluc ratios of the deletion strains to the mean of Fluc/Rluc ratio of the respective wild-type strain (same reporter derivate). In *P. putida*, the lack of TruA increased −1 frameshifting in the AD2 context ~2.2 times and UAG stop codon readthrough ~1.4 times, both effects being statistically significant (Figure 3, Table S9). In other cases, translation fidelity was similar to that observed in wild-type bacteria, even though slightly decreased in the case of UGA stop codon (0.9-fold). *P. putida* strain deficient in RluA displays comparable translational accuracy to the wild-type strain with reporters containing codons that are decoded by RluA substrate tRNAs (AD7 and UUC).

**Figure 4.**
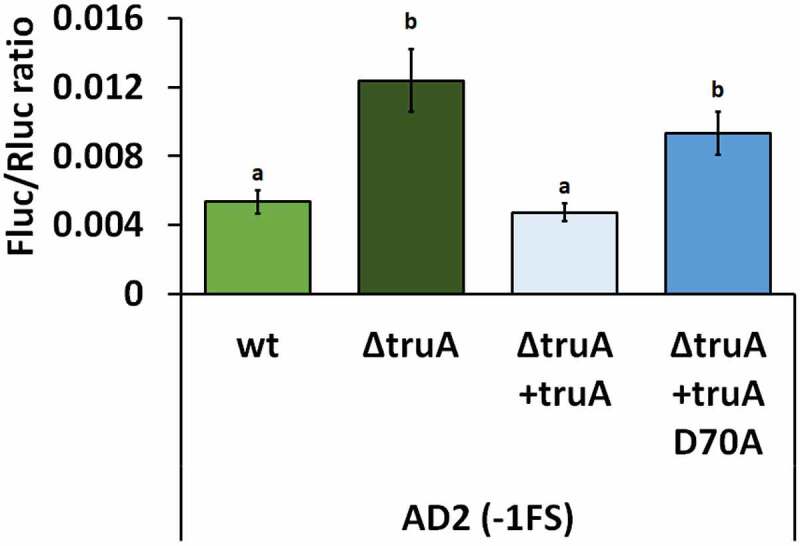
Frequency of translation errors in P. putida wild-type strain (wt), truA deletion strain (∆truA), truA deletion strain complemented with functional truA (∆truA+truA), and truA deletion strain complemented with catalytically inactive truA (∆truA+truA D70A). Letters a and b indicate homogeneity groups, different letters denote a statistically significant difference (p-value <0.001), n ≥ 12.

As catalytically inactive Ψ synthases can retain their substrate binding ability and in the case of TruB it has been shown that in *E. coli* the disruption of substrate binding ability affects the growth rate more than the disruption of pseudouridylation activity [39], we set out to determine, whether the effect on translational fidelity was due to missing pseudouridines from the tRNA or some other, possibly unknown, function of TruA. Catalytic activity of TruA has been shown to stem from aspartatic acid residue, in *P. putida* this is D70 [16,40]. When the *P. putida* ∆*truA* strain was chromosomally complemented with the functional *truA* gene (∆*truA+truA*), the −1 FS frequency at AD2 reporter was reduced, while complementation with a catalytically inactive TruA (∆*truA+truA* D70A) did not rescue frameshifting (Figure 4). Thus, high −1 FS at AD2 in *P. putida* was indeed due to the absence of Ψ-residues from the anticodon stem-loop of tRNAs.

**Figure 5.**
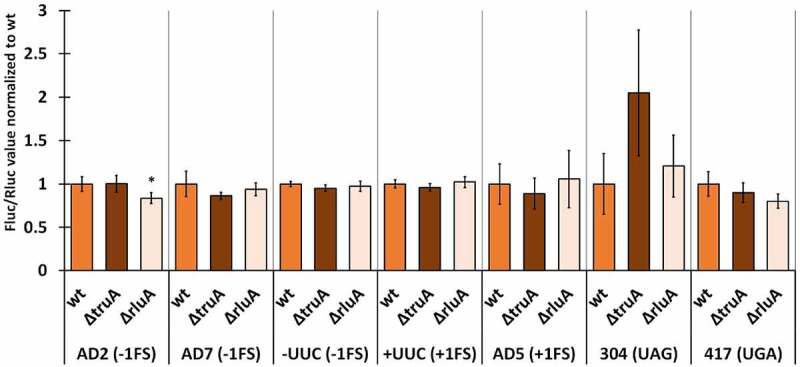
Effect of pseudouridines on translation error frequency using ΔtruA and ΔrluA strains relative to wild-type in P. aeruginosa PAO1-L. Deletion strains have been normalized against the wild-type strain within each genetic context. Error bars represent CI 95%, ‘*’ indicates p-value <0.05 compared to the respective wild-type, n ≥ 11.

In *P. aeruginosa* cells, lack of pseudouridines in the ASL had no significant effect on translational fidelity with one exception. UAG stop codon readthrough was increased in the absence of TruA, resulting in a 2-fold effect, similarly to *P. putida*. However, the effect was not statistically significant (Table S10). Lack of RluA slightly decreased (0.83-fold) −1 frameshifting on the AD2 sequence, but otherwise *ΔrluA* strain performed similarly to the wild-type *P. aeruginosa* (Figure 5).

**Figure 6.**
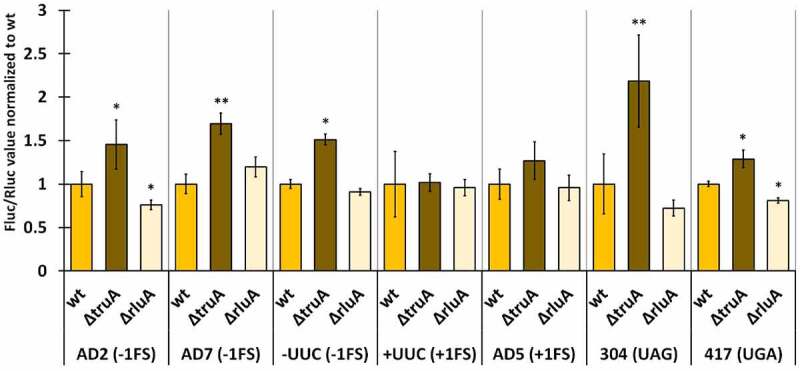
Effect of pseudouridines on translation error frequency using Δ*truA* and Δ*rluA* strains relative to wild-type in *E. coli* MG1655. Deletion strains have been normalized against the wild-type strain within each genetic context. Error bars represent CI 95%, ‘*’ indicate*s* p-value <0.05, ‘**’ indicates p-value <0.001 compared to the respective wild-type, *n ≥ 11.*

In *E. coli*, translational fidelity was affected by the lack of either pseudouridine synthase in nearly all tested reporter constructs (Figure 6, statistical significance presented in Table S11). TruA-deficient strain exhibited a ~ 1.5-fold increase in −1 frameshifting in all genetic contexts (AD2, AD7 and -UUC) while +1 frameshifting was affected in the AD5 context (1.3-fold) but not in +UUC context (Figure 6). Readthrough was also increased at both stop codons, ~2.2 times at UAG and ~1.3 times at UGA. Interestingly, RluA-deficient strain exhibited decreased frameshifting and stop codon readthrough compared to wild-type in AD2 context (0.8-fold) and both stop codons (0.7-fold – UAG; 0.8-fold – UGA). In addition to this, −1 frameshifting at AD7 sequence was increased ~1.2 times in ∆*rluA* strain in comparison to wild-type (Figure 6).

Taken together, only the increased UAG stop codon readthrough in the absence of TruA emerged as a universal characteristic when comparing the fidelity profiles between the pseudouridine-deficient strains of all three species. It was also evident that among the three species, the lack of TruA has a bigger effect on translational fidelity than the lack of RluA. Moreover, their effect was opposite, as translational errors were increased in ∆*truA* strains and decreased in ∆*rluA* strains. Lack of either pseudouridine synthase affected the translational fidelity in *E. coli* more than in *P. putida* and *P. aeruginosa*. Interestingly, on the AD2 sequence, the *Pseudomonas sp*. bacteria, which are more related and share more similarities in translational machinery and codon usage to each other than either does to *E. coli*, performed in dissimilar manner to each other, as *P. aeruginosa* was unaffected by the lack of TruA, while the translational fidelity was reduced in the TruA-deficient strains in *P. putida* and *E. coli*.

## Discussion

### Variation of mistranslation profiles between the investigated species

Programmed ribosomal frameshifting allows production of several proteins from overlapping coding sequences. It is a necessary event for propagation of some viruses [41,42], although programmed frameshift on genomic genes has also been described in prokaryotes [43,44] and eukaryotes [45]. Translocation to the new reading frame is usually directed at specific sites by secondary structures in the mRNA. Spontaneous frameshifting taking place at random locations, however, leads to synthesis of aberrant or truncated polypeptide sequences. In *E. coli* spontaneous ribosomal frameshifting as a translational error has been shown to occur at an efficiency of up to 16% at *argI* mRNA [46]. While studies regarding translational fidelity are common, they are generally focused on measuring translational error signals in a single species, thus making it difficult to assess whether the frequency of the effect is species-specific or common for other bacteria as well. Eukaryotic systems have been previously compared [47], but prokaryotic studies have centred on a single species, usually *E. coli*. Whether and to what extent translational fidelity can vary between bacterial species has not been thoroughly studied.

In the current study we show that frequency of mistranslation events, frameshifting and stop codon readthrough, are markedly different in three bacterial species. The three investigated species all belong to the class of Gammaproteobacteria but occupy different habitats in nature. *P. putida* is a soil bacterium [29], while *P. aeruginosa* (belonging to the same genus) is mostly known as an opportunistic human pathogen [48]. Facultative anaerobe *E. coli* is well-known prokaryotic model organism, commonly found in lower intestine of warm-blooded animals [49].

From our experiments it is seen that frameshifting is more frequent in *Pseudomonas* species. In particular, +1 FS is an order of magnitude higher in *P. putida* in comparison to *P. aeruginosa* and *E. coli* (Figure 2). Both −1 and +1 FS frequency at UUC slippery site is 2–7 times higher in *Pseudomonas* species than in *E. coli*. Stop codon readthrough, on the other hand, is more frequent in *E. coli*. One reason behind this variation in error frequency between bacterial species can be modification pattern of tRNA around ASL. The results of these studies will be discussed below.

UGA translational readthrough is a known phenomenon in *E. coli*, occurring at a frequency of up to 20% [50], which is consistent with our experiments where we measured the readthrough efficiency at 12% (Figure 2). During UGA stop codon readthrough either tryptophan or selenocysteine is incorporated into the growing polypeptide chain. As a SECIS element required for the selenocysteine recoding event is not present in our Rluc-Fluc mRNA, we can presume that UGA stop codon is likely to be suppressed by tRNA^Trp^. In *E. coli*, the tRNA^Trp^ is hypermodified at the position 37 by enzymes MiaA (A to i^6^A) and MiaB (i^6^A to ms^2^i^6^A) [51]. In *P. putida* enzyme MiaE has been identified, which further modifies ms^2^i^6^A to ms^2^io^6^A [52]. Based on genome annotation, MiaE is also present in *P. aeruginosa* (strain PAO-1) [53], but absent from *E. coli* (strain K-12) [54]. tRNA position 37 modifications have been shown to affect readthrough as lack of MiaB decreases UGA readthrough in *E. coli* [55]. Therefore, it is possible that the significant difference regarding UGA readthrough in our experiments between *E. coli* (12%) and *Pseudomonas sp*. (0.08% in *P. putida*, 0.04% in *P. aeruginosa*) could be due to differences in modifications at the position 37 of tRNA^Trp^ in *Pseudomonas sp*. and *E. coli* (Figure 2). Based on our results, UAG stop codon is suppressed at a lower rate than UGA in all of the investigated species, which is consistent with what has been previously reported in *E. coli* [50].

### Effect of Ψ32/38–40 on mistranslation

Pseudouridines in the ASL of tRNAs have been previously shown to affect reading frame maintenance during translation. Interestingly, while +1 frameshifting was increased in *Salmonella enterica* serovar Typhimurium cells lacking Ψ38–40 [10,56], in *S. cerevisiae* stop codon readthrough and +1 frameshifting were decreased in cells missing Ψ38–39 [57]. In our experiments, lack of Ψ residues in the ASL affects translational frameshifting in three bacterial species in a different way (Figures 3, 5 and 6). Specifically, −1 FS frequency in the AD2 context in ∆*truA* strains is increased 2.2-fold in *P. putida* and 1.5-fold in *E. coli*. ∆*truA* strain of *E. coli* has elevated −1 FS frequency in all three reporters. *P. putida* ∆*truA* strain has increased −1 FS only in AD2 context and in *P. aeruginosa* the absence of TruA does not seem to affect −1 FS frequency at all. It is important to note that wild-type *E. coli* has a lower level −1 FS as compared to *Pseudomonas* species. Absence of Ψ residues does not cause altered +1 frameshifting in any of the investigated bacterial species, which is different from what has been reported in *S. enterica* strain missing Ψ38–40 [10] demonstrating importance of the specific sequence context. There is no simple explanation why −1 FS frequency depends on the presence of Ψ residues at positions 38–40 of ASL. Structural studies on the *E. coli* 70S ribosomes in complex with tRNA^SufA6^ (a frameshift suppressor derivative of tRNA^Pro^) have revealed ASL conformational change during +1 frameshifting. Interaction between tRNA bases 32 and 38 is lost in the ribosomal P and E sites [26]. Note that uridines 32 and 38 are substrates for RluA and TruA, respectively. Moreover, tRNA^SufA6^ in the P site shows extensive conformational rearrangements of the 30S head and body domains [26]. Our results on the FS frequency in TruA defective strains suggest that ASL conformational change can occur also during −1 FS event. If this is true, pseudouridine-dependent conformational rigidity helps to keep the reading frame during translation by preventing conformational change in ASL. We cannot exclude a possibility that the ribosomes of different bacterial species use alternative ways to keep mRNA reading frame during translation.

Regarding the changes in translational fidelity in Ψ-synthase-deficient strains, we noticed that UAG stop codon readthrough was enhanced in ∆*truA* strains in all three bacterial species (Figures 3, 5 and 6). Previously it has been shown that glutamine is incorporated at the UAG codon during mistranslation [58]. There are two glutamine tRNAs in *E. coli* (with anticodons CUG and UUG) and a single tRNA in *P. putida* and *P. aeruginosa* (anticodon UUG) and they are all targets for the pseudouridine synthase TruA [59]. More specifically, the U at position 38 in tRNA^Gln^ is isomerized. Our results indicate that a Ψ38 helps to make the decoding stricter, i.e. decrease the frequency of stop codon readthrough. Similar effect of Ψ deficiency at position 38 was previously shown to decrease mistranslation at His codons [20]. tRNA^Gln^ with the anticodon CUG, that normally decodes CAG codon can base pair with the UAG stop codon if a G–U wobble base pair is formed between the first nucleotide in the stop codon and the third nucleotide in the anticodon. This is also required for the tRNA with the anticodon UUG, which would additionally require non-canonical base pairing between the last G of the stop codon and the first U of anticodon. It is likely that a Ψ38 stabilizes the structure of the anticodon stem-loop due to stronger stacking interactions and thereby reducing conformational flexibility. Stable ASL structure would prevent conformational flexibility required for the frameshift event. With U38, the stem-loop allows for more flexibility leading to increased frequency of mistranslation events.

### Concluding remarks

Based on the results obtained we make the following conclusions:

1. Frameshift frequency at known slippery sequence sites varies in *Pseudomonas* species and in *E. coli* up to 10 times. General order of FS frequency is *P.putida* > *P. aeruginosa* > *E. coli*.
2. FS frequency in both directions at UUU UUC site is 3 times more frequent in *Pseudomonas* species as compared to *E. coli*.
3. Stop codon readthrough at UAG codon is similar in the three bacterial species. In contrast, readthrough at UGA is three orders of magnitude more frequent in *E. coli* as compared to *Pseudomonas* species.
4. TruA supports reading frame maintenance and reduces stop codon readthrough in *E. coli*. In *P. putida*, TruA has a context-specific effect on −1 frameshifting and decreases misreading at UAG. In *P. aeruginosa* TruA does not appear to affect translation fidelity.

## Materials and methods

### Bacterial strains, plasmids, and media

The bacterial strains and plasmids used in this study are listed in Table 1. All *P. putida* strains are derivatives of PaW85 which is isogenic to KT2440 [60,61], *P. aeruginosa* strains are derivatives of PAO1-L [62], and *E. coli* strains are derivatives of MG1655 [63]. *P. putida* strains were grown at temperature 30°C, *E. coli* and *P. aeruginosa* strains were grown at 37°C, except for measurement of translational fidelity using the Rluc-Fluc system, when all the cells were grown at 30°C.

**Table 1.** Bacterial strains and plasmids used in this study.

Strain or plasmid	Description	Source
*P. putida*		
PaW85	Wild-type, isogenic to KT2440	[60,61]
PaW ∆truA	PaW85, Δ*truA* (PP1994)	[34]
PaW ∆rluA	PaW85, Δ*rluA* (PP1731)	[16]
PaW ΔtruA + truA	PaW85, ΔtruA strain containing lacI-Ptac-truA gene cassette with functional truA gene in the intergenic region between glmS and PP5408 (Gm^R^)	[16]
PaW ΔtruA + truA D70A	PaW85, ΔtruA strain containing lacI-PtactruA D70A gene cassette in the intergenic region between glmS and PP5408 (Gm^R^). The catalytic aspartic acid of TruA is mutated to alanine.	[16]
*P. aeruginosa*		
PAO-1 L	Wild-type, PAO1 subline, University of Lausanne, Dieter Haas collection	Stephan Heeb
PAO-1 L ∆truA	PAO1-L, Δ*truA* (PA3114)	[16]
PAO-1 L ∆rluA	PAO1-L, *ΔrluA* (PA3246)	[16]
*E. coli*		
MG1655	Wild-type	[63]
BW25113 ΔtruA	From Keio collection, donor of *ΔtruA::km* for the construction of MG ΔtruA	[18]
BW25113 ΔrluA	From Keio collection, donor of *ΔrluA::km* for the construction of MG ΔrluA	[18]
MG ∆truA	MG1655, Δ*truA*	This study
MG ∆rluA	MG1655, Δ*rluA*	This study
Plasmids		
pSEVA/lacItac	Plasmid carrying lacI-P_tac_ cassette	[16]
pSEVA RFwt	Reporter plasmid without any frameshifts or premature stop codons, Km^R^ + Gm^R^	This study
pSEVA AD2	Reporter plasmid with a − 1 frameshift signal between the Rluc and Fluc gene, Km^R^ + Gm^R^	This study
pSEVA AD5	Reporter plasmid with a + 1 frameshift signal between the Rluc and Fluc gene, Km^R^ + Gm^R^	This study
pSEVA AD7	Reporter plasmid with a − 1 frameshift signal between the Rluc and Fluc gene, Km^R^ + Gm^R^	This study
pSEVA 304 UAG	Reporter plasmid with a premature stop codon (UAG) in the Fluc gene, Km^R^ + Gm^R^	This study
pSEVA 417 UGA	Reporter plasmid with a premature stop codon (UGA) in the Fluc gene, Km^R^ + Gm^R^	This study
pSEVA RF NcoI/PstI	Reporter plasmid with NcoI and PstI sites in the linker region between Rluc and Fluc genes, Km^R^ + Gm^R^	This study
pSEVA UUC-	Reporter plasmid with a slippery sequence and a − 1 frameshift signal between the Rluc and Fluc gene, Km^R^ + Gm^R^	This study
pSEVA UUC+	Reporter plasmid with a slippery sequence and a + 1 frameshift signal between the Rluc and Fluc gene, Km^R^ + Gm^R^	This study

For complete medium either LB, or glc + CAA was used. For glc + CAA M9 buffer was supplemented with casamino acids (CAA) with tryptone and glucose both at final concentration 0.2%. Solid medium contained 1.5% Difco agar. Antibiotics were added at final concentrations: Kanamycin (Km) 50 µg/mL, gentamycin (Gm) 10 µg/mL.

### Construction of strains and plasmids

For construction of MG1655 TruA and RluA deletion strains, BWΔ*truA::km* and BWΔ*rluA::km* strains were obtained from the Keio collection [18]. MG1655 deletion strains were generated by transferring the Δ*truA::km* or Δ*rluA::km* fragment into MG1655 by P1 phage transduction. In the next step, kanamycin resistant colonies were selected, and the kanamycin resistance gene was removed by expressing flippase from a temperature sensitive plasmid pCP20 at 30°C, which was afterwards removed from cells by cultivation at 37°C [64]. Successful deletion was confirmed by PCR.

Broad-host range plasmid pSEVA/lacItac [16] was chosen as carrier for the dual-luciferase assay system. Frameshift reporters AD2, AD5 and AD7, and stop codon readthrough reporters 304UAG and 417UGA were based on previously published reporters [38], transferred under the control of the Tac-promoter of pSEVA/lacItac by circular polymerase extension cloning [65]. To generate UUC ±FS reporters, we first constructed pSEVA RF NcoI/PstI plasmid that allows cloning of wide range of sequences between the *rluc* and *fluc* genes. Site directed mutagenesis and Eco81I and MluI fragment replacement on the initial plasmid (pSEVA RFwt) were used to generate NcoI and PstI restriction sites to the ends of the linker region of the Rluc and Fluc. Oligonucleotides containing the UUC sequence were designed with NcoI and PstI sites in the flanking regions. Then, the oligonucleotides were annealed, cleaved with NcoI and PstI and inserted into the pSEVA RF NcoI/PstI plasmid.

### Dual luciferase translation assay

Cells carrying the reporter were grown overnight at 30°C in 1.5 mL glc + CAA medium supplemented with either kanamycin (50 μg/mL for *P.putida* and *E.coli*) or gentamycin (10 μg/mL for *P.aeruginosa*). Cultures were diluted to OD_580_ ~ 0.1 into fresh glc + CAA medium containing the appropriate antibiotic and IPTG (0.5 mM). Cells carrying frameshift reporters were grown in 2 ml and cells carrying stop codon readthrough reporters were grown in 4 mL aliquots. After 3 hours of growth at 30°C, cells were collected, pelleted and flash frozen in liquid nitrogen. Frozen cells were resuspended in 400 μL of Passive Lysis buffer of Dual-Luciferase Reporter Assay System (Promega) and placed on ice for 10 min.

For measurement, 50 μL of cell extract was assayed for Fluc activity; the reaction mixture was diluted 50-fold using Passive Lysis buffer and 50 μL of the diluted reaction mixture was assayed for Rluc activity. Luminescence was measured using a TECAN Infinite ProM200 plate reader. Fluc activity was measured over a 10 second interval 2 minutes after starting the reaction, Rluc activity was measured over a 10 second interval 4 minutes after Fluc activity was measured. Raw values of luciferase assays are shown in the Table S12. Taking the dilution factor into account, the ratio of Fluc activity to Rluc activity was calculated and the Fluc/Rluc ratio of each replicate was normalized against the mean value of the reporter system, where both luciferase genes were functional and in the same frame (pSEVA RFwt). The normalization standard (Fluc/Rluc ratio using pSEVA RFwt construct) was determined separately in the wild-type strain for each species.

### Statistical analysis

Shapiro-Wilk test was used to determine the normality of the dataset. As the data did not follow normal distribution, non-parametric method was used to compare different datasets. Kruskal-Wallis test was performed, followed by Dunn’s post-hoc test. Calculations were performed using Statistica software (TIBCO Software).

## Supplementary Material

Supplemental Material

## Data Availability

The authors confirm that the data supporting the findings of this study are available within the article and its supplementary materials.
