# Supplementary material for: Variance in translational fidelity of different bacterial species is affected by pseudouridines in the tRNA anticodon stem-loop
Source: RNA Biol. 2022 Sep 11;19(1):1050–8. doi: 10.1080/15476286.2022.2121447 (PMC9481147; doi:10.1080/15476286.2022.2121447)
Supplement: Supplemental Material [file KRNB_A_2121447_SM7367.docx]

Supplementary material

**Table S1.** Targets of TruA and RluA enzymes within the tRNAs decoding the linker-region of the WT sequence of the Rluc-Fluc translation assay system. T denotes tRNA modified by TruA, R denotes tRNA modified by RluA. * indicates that tRNA decoding a synonymous codon is modified by the corresponding enzyme.

| **WT sequence (no frameshift)** | CAA | ATG | TCG | ACG | GGG | GCC | CCT^a^ | AGG | AGA | TCT^b^ | AGC | |
| --- | --- | --- | --- | --- | --- | --- | --- | --- | --- | --- | --- | --- |
| Amino acid | Q | M | S | T | G | A | P | R | R | S | S | |
| *P. putida* PaW85 | T | T | T, R |  |  | T | T* |  |  | T*,R* |  |  |
| *P. aeruginosa* PAO1-L | T | T | T, R | T |  | T | T* |  |  | T*,R* |  |  |
| *E. coli* MG1655 | T | T |  | T |  | T | T* | T* | T | T* | T* |  |

^a^ – tRNA^Pro(AGG)^ not present in any of the strains, tRNA^Pro(GGG)^ modified by TruA

^b^ – tRNA^Ser(AGA)^ not present in any of the strains, tRNA^Ser(GGA)^ modified by TruA in all strains, tRNA^Ser(CGA)^ modified by both TruA and RluA in PaW85 and PAO1-L

**Table S2.** Targets of TruA and RluA enzymes within the tRNAs decoding the linker-region of the AD2 sequence of the Rluc-Fluc translation assay system. T denotes tRNA modified by TruA, R denotes tRNA modified by RluA. * indicates that tRNA decoding a synonymous codon is modified by the corresponding enzyme. Red letters indicates difference from the initial sequence and orange background denotes shifted reading frame.

| **AD2 (-1 frameshift)** | CAA | ATG | TCG | ∆A | CGG | GGG | CCC | CTA | GGA | GAT | CTA | GCG^a^ | |
| --- | --- | --- | --- | --- | --- | --- | --- | --- | --- | --- | --- | --- | --- |
| Amino acid | Q | M | S |  | R | G | P | L | G | D | L | A | |
| *P. putida* PaW85 | T | T | T, R |  |  |  | T | T |  |  | T | T* |  |
| *P. aeruginosa* PAO1-L | T | T | T, R |  | T* |  | T | T |  |  | T | T* |  |
| *E. coli* MG1655 | T | T |  |  |  |  | T | T |  |  | T | T* |  |

^a^ – tRNA^Ala(CGC)^ not present in any of the strains, tRNA^Ala(GGC)^ modified by TruA

**Table S3.** Targets of TruA and RluA enzymes within the tRNAs decoding the linker-region of the AD7 sequence of the Rluc-Fluc translation assay system. T denotes tRNA modified by TruA, R denotes tRNA modified by RluA. * indicates that tRNA decoding a synonymous codon is modified by the corresponding enzyme. Red letters indicate difference from the initial sequence and orange background denotes shifted reading frame.

| **AD7 (-1 frameshift)** | CAA | ATG | TCG | AGC | TTG | GGA | TAA | GGA | GAT | CTA | GCG^a^ | |
| --- | --- | --- | --- | --- | --- | --- | --- | --- | --- | --- | --- | --- |
| Amino acid | Q | M | S | S | L | G | stop | G | D | L | A | |
| *P. putida* PaW85 | T | T | T, R |  | T, R |  |  |  |  | T | T* |  |
| *P. aeruginosa* PAO1-L | T | T | T, R |  | T, R |  |  |  |  | T | T* |  |
| *E. coli* MG1655 | T | T |  | T* | T, R |  |  |  |  | T | T* |  |

^a^ – tRNA^Ala(CGC)^ not present in any of the strains, tRNA^Ala(GGC)^ modified by TruA

**Table S4.** Targets of TruA and RluA enzymes within the tRNAs decoding the linker-region of the -UUC sequence of the Rluc-Fluc translation assay system. T denotes tRNA modified by TruA, R denotes tRNA modified by RluA. * indicates that tRNA decoding a synonymous codon is modified by the corresponding enzyme. Red letters indicate difference from the initial sequence and orange background denotes shifted reading frame.

| **-UUC (-1 frameshift)** | CAA | ATG | CTG | CAG^a^ | GGG | CCT^b^ | TTT^c^ | TTT^c^ | CTC | CTA | GCG^d^ | CTG | GAT | CCC |
| --- | --- | --- | --- | --- | --- | --- | --- | --- | --- | --- | --- | --- | --- | --- |
| Amino acid | Q | M | L | Q | G | P | F | F | L | L | A | L | D | P |
| *P. putida* PaW85 | T | T | T | T* |  | T* | T*,R* | T*,R* | T | T | T* | T |  | T |
| *P. aeruginosa* PAO1-L | T | T | T | T* |  | T* | T*,R* | T*,R* | T | T | T* | T |  | T |
| *E. coli* MG1655 | T | T | T | T |  | T* | T*,R* | T*,R* | T | T | T* | T |  | T |

^a^ – tRNA^Gln(CUG)^ not present in PaW85 and PAO1-L, tRNA^Gln(CUU)^ modified by TruA

^b^ – tRNA^Pro(AGG)^ not present in any of the strains, tRNA^Pro(GGG)^ modified by TruA

^c^ – tRNA^Phe(AAA)^ not present in any of the strains, tRNA^Phe(GAA)^ modified by TruA

^d^ – tRNA^Ala(CGC)^ not present in any of the strains, tRNA^Ala(GGC)^ modified by TruA

**Table S5.** Targets of TruA and RluA enzymes within the tRNAs decoding the linker-region of the AD5 sequence of the Rluc-Fluc translation assay system. T denotes tRNA modified by TruA, R denotes tRNA modified by RluA. * indicates that tRNA decoding a synonymous codon is modified by the corresponding enzyme.

| **AD5 (+1 frameshift)** | CAA | ATG | TCG | ACG | GGG | GCC | CCT^a^ | AGG | +AAG | ATC | | TAG | |  |
| --- | --- | --- | --- | --- | --- | --- | --- | --- | --- | --- | --- | --- | --- | --- |
| Amino acid | Q | M | S | T | G | A | P | R | K | I | | stop | |  |
| *P. putida* PaW85 | T | T | T, R |  |  | T | T* |  |  | |  | |  | |
| *P. aeruginosa* PAO1-L | T | T | T, R | T |  | T | T* |  |  | |  | |  | |
| *E. coli* MG1655 | T | T |  | T |  | T | T* | T* |  | |  | |  | |

^a^ – tRNA^Pro(AGG)^ not present in any of the strains, tRNA^Pro(GGG)^ modified by TruA

**Table S6.** Targets of TruA and RluA enzymes within the tRNAs decoding the linker-region of the +UUC sequence of the Rluc-Fluc translation assay system. T denotes tRNA modified by TruA, R denotes tRNA modified by RluA. * indicates that tRNA decoding a synonymous codon is modified by the corresponding enzyme.

| **+UUC (+1 frameshift)** | CAA | ATG | CTG | CAG^a^ | GGG | CCT^b^ | TTT^c^ | TTT^c^ | CTC | CTA | GCG^d^ | AAC | TGG | ATC |
| --- | --- | --- | --- | --- | --- | --- | --- | --- | --- | --- | --- | --- | --- | --- |
| Amino acid | Q | M | L | Q | G | P | F | F | L | L | A | L | D | P |
| *P. putida* PaW85 | T | T | T | T* |  | T* | T*,R* | T*,R* | T | T | T* | T |  | T |
| *P. aeruginosa* PAO1-L | T | T | T | T* |  | T* | T*,R* | T*,R* | T | T | T* | T |  | T |
| *E. coli* MG1655 | T | T | T | T |  | T* | T*,R* | T*,R* | T | T | T* | T |  | T |

^a^ – tRNA^Gln(CUG)^ not present in PaW85 and PAO1-L, tRNA^Gln(CUU)^ modified by TruA

^b^ – tRNA^Pro(AGG)^ not present in any of the strains, tRNA^Pro(GGG)^ modified by TruA

^c^ – tRNA^Phe(AAA)^ not present in any of the strains, tRNA^Phe(GAA)^ modified by TruA

^d^ – tRNA^Ala(CGC)^ not present in any of the strains, tRNA^Ala(GGC)^ modified by TruA

**Table S7.** Targets of TruA enzyme within the tRNAs decoding the codons 303-305 of the fluc gene in the Rluc-Fluc translation assay system. T denotes tRNAs modified by TruA.

| Codon number | 303 | 304 | 305 |
| --- | --- | --- | --- |
| **304 UAG (stop codon readthrough)** | AAA | TAG | GAT |
| Amino acid | K | stop | D |
| *P. putida* PaW85 |  | T ^a^ |  |
| *P. aeruginosa* PAO1-L |  | T ^a^ |  |
| *E. coli* MG1655 |  | T ^a^ |  |

^a^ – Based on previous studies (performed in *E. coli*) tRNA^Gln(UUG)^ (present in all strains), tRNA^Gln(CUG)^ (present in MG1655) are the most likely suppressors of TAG stop codon (Nilsson and Rydén-Aulin 2003). Both are modified by TruA.

**Table S8.** Targets of TruA enzyme within the tRNAs decoding the codons 416-418 of the fluc gene in the Rluc-Fluc translation assay system. T denotes tRNAs modified by TruA.

| Codon number | 416 | 417 | 418 |
| --- | --- | --- | --- |
| **417 UGA (stop codon readthrough)** | GGA | TGA | CTA |
| Amino acid | G | stop | L |
| *P. putida* PaW85 |  |  | T |
| *P. aeruginosa* PAO1-L |  |  | T |
| *E. coli* MG1655 |  |  | T |

**Table S9.** Statistical analysis of the values of the dual-luciferase assay in P. putida PaW85 wild-type, ΔtruA, ΔrluA, ΔtruA+truA and ΔtruA+truA D70A strains. P-values of the non-parametric Kruskal-Wallis test are presented, statistically significant values are in bold.

| **AD2 (-1FS)** | wt | Δ*truA* | Δ*rluA* | Δ*truA+truA* | Δ*truA+truA* D70A |
| --- | --- | --- | --- | --- | --- |
| wt |  | **<0.001** | 1.0 | 1.0 | **0.0096** |
| Δ*truA* | **<0.001** |  | **<0.001** | **<0.001** | 1.0 |
| Δ*rluA* | 1.0 | **<0.001** |  | 1.0 | **0.003** |
| ∆*truA+truA* | 1.0 | **<0.001** | 1.0 |  | **<0.001** |
| ∆*truA+*truA D70A | **0.0096** | 1.0 | **0.003** | **<0.001** |  |
| **AD7 (-1FS)** | wt | Δ*truA* | Δ*rluA* |  |  |
| wt |  | 0.591 | 1.0 |  |  |
| Δ*truA* | 0.591 |  | 0.769 |  |  |
| Δ*rluA* | 1.0 | 0.769 |  |  |  |
| **-UUC (-1FS)** | wt | Δ*truA* | Δ*rluA* |  |  |
| wt |  | 0.126 | 0.735 |  |  |
| Δ*truA* | 0.126 |  | 1.0 |  |  |
| Δ*rluA* | 0.735 | 1.0 |  |  |  |
| **AD5 (+1FS)** | wt | Δ*truA* | Δ*rluA* |  |  |
| wt |  | 1.0 | 1.0 |  |  |
| Δ*truA* | 1.0 |  | 1.0 |  |  |
| Δ*rluA* | 1.0 | 1.0 |  |  |  |
| **+UUC (+1FS)** | wt | Δ*truA* | Δ*rluA* |  |  |
| wt |  | 1.0 | 0.562 |  |  |
| Δ*truA* | 1.0 |  | 1.0 |  |  |
| Δ*rluA* | 0.562 | 1.0 |  |  |  |
| **304 (UAG)** | wt | Δ*truA* | Δ*rluA* |  |  |
| wt |  | **<0.001** | 1.0 |  |  |
| Δ*truA* | **<0.001** |  | **<0.001** |  |  |
| Δ*rluA* | 1.0 | **<0.001** |  |  |  |
| **417 (UGA)** | wt | Δ*truA* | Δ*rluA* |  |  |
| wt |  | **0.017** | 1.0 |  |  |
| Δ*truA* | **0.017** |  | 0.083 |  |  |
| Δ*rluA* | 1.0 | 0.083 |  |  |  |

**Table S10.** Statistical analysis of the values of the dual-luciferase assay in P. aeruginosa PAO1-L wild-type, ΔtruA and ΔrluA strains. P-values of the non-parametric Kruskal-Wallis test are presented, statistically significant values are in bold.

| **AD2 (-1FS)** | wt | Δ*truA* | Δ*rluA* |
| --- | --- | --- | --- |
| wt |  | 1.0 | **0.005** |
| Δ*truA* | 1.0 |  | **0.004** |
| Δ*rluA* | **0.005** | **0.004** |  |
| **AD7 (-1FS)** | wt | Δ*truA* | Δ*rluA* |
| wt |  | 0.127 | 1.0 |
| Δ*truA* | 0.127 |  | 0.160 |
| Δ*rluA* | 1.0 | 0.160 |  |
| **-UUC (-1FS)** | wt | Δ*truA* | Δ*rluA* |
| wt |  | 0.159 | 0.407 |
| Δ*truA* | 0.159 |  | 1.0 |
| Δ*rluA* | 0.407 | 1.0 |  |
| **AD5 (+1FS)** | wt | Δ*truA* | Δ*rluA* |
| wt |  | 1.0 | 1.0 |
| Δ*truA* | 1.0 |  | 1.0 |
| Δ*rluA* | 1.0 | 1.0 |  |
| **+UUC (+1FS)** | wt | Δ*truA* | Δ*rluA* |
| wt |  | 0.735 | 1.0 |
| Δ*truA* | 0.735 |  | 0.276 |
| Δ*rluA* | 1.0 | 0.276 |  |
| **304 (UAG)** | wt | Δ*truA* | Δ*rluA* |
| wt |  | 0.111 | 1.0 |
| Δ*truA* | 0.111 |  | 0.429 |
| Δ*rluA* | 1.0 | 0.429 |  |
| **417 (UGA)** | wt | Δ*truA* | Δ*rluA* |
| wt |  | 1.0 | 0.095 |
| Δ*truA* | 1.0 |  | 0.603 |
| Δ*rluA* | 0.095 | 0.603 |  |

**Table S11.** Statistical analysis of the values of the dual-luciferase assay in Escherichia coli MG1655 wild-type, ΔtruA and ΔrluA strains. P-values of the non-parametric Kruskal-Wallis test are presented, statistically significant values are in bold.

| **AD2 (-1FS)** | wt | Δ*truA* | Δ*rluA* |
| --- | --- | --- | --- |
| wt |  | **0.046** | **0.047** |
| Δ*truA* | **0.046** |  | **<0.001** |
| Δ*rluA* | **0.047** | **<0.001** |  |
| **AD7 (-1FS)** | wt | Δ*truA* | Δ*rluA* |
| wt |  | **<0.001** | 0.294 |
| Δ*truA* | **<0.001** |  | **<0.001** |
| Δ*rluA* | 0.294 | **<0.001** |  |
| **-UUC (-1FS)** | wt | Δ*truA* | Δ*rluA* |
| wt |  | **0.003** | 0.244 |
| Δ*truA* | **0.003** |  | **<0.001** |
| Δ*rluA* | 0.244 | **<0.001** |  |
| **AD5 (+1FS)** | wt | Δ*truA* | Δ*rluA* |
| wt |  | 0.187 | 1.0 |
| Δ*truA* | 0.187 |  | **0.027** |
| Δ*rluA* | 1.0 | **0.027** |  |
| **+UUC (+1FS)** | wt | Δ*truA* | Δ*rluA* |
| wt |  | 0.056 | 0.263 |
| Δ*truA* | 0.056 |  | 1.0 |
| Δ*rluA* | 0.263 | 1.0 |  |
| **304 (UAG)** | wt | Δ*truA* | Δ*rluA* |
| wt |  | **<0.001** | 1.0 |
| Δ*truA* | **<0.001** |  | **<0.001** |
| Δ*rluA* | 1.0 | **<0.001** |  |
| **417 (UGA)** | wt | Δ*truA* | Δ*rluA* |
| wt |  | **0.035** | **0.001** |
| Δ*truA* | **0.035** |  | **<0.001** |
| Δ*rluA* | **0.001** | **<0.001** |  |

**References**

Nilsson, M. and M. Rydén-Aulin (2003). "Glutamine is incorporated at the nonsense codons UAG and UAA in a suppressor-free *Escherichia coli* strain." Biochimica et Biophysica Acta (BBA)-Gene Structure and Expression **1627**(1): 1-6.
